# Pyrethroid and Carbamate Resistance in *Anopheles funestus* Giles along Lake Kariba in Southern Zambia

**DOI:** 10.4269/ajtmh.19-0664

**Published:** 2020-07-02

**Authors:** Javan Chanda, Kochelani Saili, Foustina Phiri, Jennifer C. Stevenson, Mulenga Mwenda, Sandra Chishimba, Conceptor Mulube, Brenda Mambwe, Christopher Lungu, Duncan Earle, Adam Bennett, Thomas P. Eisele, Mulakwa Kamuliwo, Richard W. Steketee, Joseph Keating, John M. Miller, Chadwick H. Sikaala

**Affiliations:** 1 PATH Malaria Control and Elimination Partnership in Africa (MACEPA), Lusaka, Zambia;; 2 National Malaria Elimination Centre, Zambia Ministry of Health, Lusaka, Zambia;; 3 Macha Research Trust, Choma, Zambia;; 4 W. Harry Feinstone Department of Molecular Microbiology and Immunology, Johns Hopkins Bloomberg School of Public Health, Baltimore, Maryland;; 5 Malaria Elimination Initiative, Global Health Group, University of California San Francisco, San Francisco, California;; 6 Department of Tropical Medicine, Center for Applied Malaria Research and Evaluation, Tulane University School of Public Health and Tropical Medicine, New Orleans, Louisiana;; 7 PATH MACEPA, Seattle, Washington;; 8 SADC Malaria Elimination Eight Secretariat, Windhoek, Namibia

## Abstract

Whereas data on insecticide resistance and its underlying mechanisms exist for parts of Zambia, data remain limited in the southern part of the country. This study investigated the status of insecticide resistance, metabolic mechanisms, and parasite infection in *Anopheles funestus* along Lake Kariba in southern Zambia. Indoor-resting mosquitoes were collected from 20 randomly selected houses within clusters where a mass drug administration trial was conducted and raised to F1 progeny. Non–blood-fed 2- to 5-day-old female *An. funestus* were exposed to WHO insecticide-impregnated papers with 0.05% deltamethrin, 0.1% bendiocarb, 0.25% pirimiphos-methyl, or 4% dichloro-diphenyl-trichloroethane (DDT). In separate assays, *An. funestus* were pre-exposed to piperonyl butoxide (PBO) to determine the presence of monooxygenases. Wild-caught *An. funestus* that had laid eggs for susceptibility assays were screened for circumsporozoite protein of *Plasmodium falciparum* by ELISA, and sibling species were identified by polymerase chain reaction. *Anopheles funestus* showed resistance to deltamethrin and bendiocarb but remained susceptible to pirimiphos-methyl and DDT. The pre-exposure of *An. funestus* to PBO restored full susceptibility to deltamethrin but not to bendiocarb. The overall sporozoite infection rate in *An. funestus* populations was 5.8%. Detection of pyrethroid and carbamate resistance in *An. funestus* calls for increased insecticide resistance monitoring to guide planning and selection of effective insecticide resistance management strategies. To prevent the development of resistance and reduce the underlying vectorial capacity of mosquitoes in areas targeted for malaria elimination, an effective integrated vector management strategy is needed.

## INTRODUCTION

Over the last 15 years, long-lasting insecticidal nets (LLINs), indoor residual spraying (IRS), intermittent preventive treatment, prompt diagnosis, and treatment with artemisinin-based combination therapy have been scaled-up for malaria control and elimination in sub-Saharan Africa, reducing malaria incidence by 22% and mortality by 29% between 2010 and 2018.^1^ Equally in Zambia, malaria incidence declined by 5% and mortality by 55% between 2010 and 2018.^2^ Most of the gains reported in malaria reduction in sub-Saharan Africa are attributed to the rapid scale-up of vector control interventions.^3^ However, the emergence of insecticide resistance in major malaria vectors puts these fragile gains at risk.^4,5^

In Zambia, *Plasmodium falciparum* account for 98% of all malaria cases reported at health facilities.^2^ Malaria transmission is maintained by three major vectors: *An. gambiae sensu stricto* Giles, *Anopheles arabiensis* Paton, and *Anopheles funestus sensu stricto* Giles.^6^ To effectively control these malaria vectors and reduce malaria transmission, the Ministry of Health (MoH) through the National Malaria Control Programme has scaled-up LLINs and IRS for malaria control and elimination in the country. Operational vector control with LLINs and IRS in Zambia depends on five classes of insecticides recommended by the WHO^7^: pyrethroids, carbamates, organophosphates, organochlorines, and neonicotinoids. Of these insecticides, pyrethroids, are the only insecticide class currently the WHO recommended for bednet impregnation because of their effectiveness, low toxicity to humans, and high excito-repellent effects on mosquitoes.^8^ A new-generation mixture LLIN called Interceptor^®^ G2 (BASF^©^, Ludwigshafen, Germany) that combines chlorfenapyr (a pyrrole) and alphacypermethrin (a pyrethroid) has been developed by BASF^©^ (Ludwigshafen, Germany) and prequalifications listed by the WHO^9^ but has yet to be rolled out widely. Entomological studies conducted in Zambia and Tanzania have demonstrated the impact of using either LLINs or IRS on reducing malaria vector abundance, infection rates, and malaria transmission.^10–12^ Nonetheless, the effectiveness of LLINs and IRS depends on high coverage within the community, mosquito susceptibility to insecticides used, and indoor-biting and resting behaviors of mosquitoes.

Long-lasting insecticidal nets and IRS exploit the biting and resting behaviors of local malaria vectors to reduce malaria transmission. Long-lasting insecticidal nets are designed to reduce human–vector contact by targeting night-biting mosquitoes, whereas IRS aims to reduce the life span of indoor-biting (endophagic) and indoor-resting (endophilic) mosquitoes.^13^ The endophagic and endophilic behavioral characteristics of mosquitoes tend to expose them to insecticides through contact with LLINs and or IRS.^14^ In addition, the same insecticides used for public health are equally used in agriculture for pest control and usually end up contaminating mosquito-breeding sites.^15^ In this regard, there has been increased selection pressure on mosquito populations that demonstrate either physiological or behavioral resistance to different classes of insecticides. The common mechanisms responsible for physiological resistance are metabolic detoxification and decreased target-site sensitivity.^16^ In metabolic detoxification, the insecticide is prevented from reaching the site of action in lethal concentration by detoxifying enzyme groups (P450s monooxygenases, glutathione s-transferases, and esterases).^16^ By contrast, decreased target-site sensitivity mechanism reduces the rate at which the insecticide binds to its target site (knockdown resistance [*kdr*], dieldrin resistance [*rdl*], and acetylcholinesterase [*ace.1*]).^16–18^ The other mechanisms of resistance include penetration resistance which occurs as a result of cuticle thickening^19^ and behavioral resistance where mosquitoes may evolve to avoid insecticidal contact in malaria vectors.^20,21^

Following a decade of scaling-up LLINs and IRS in Zambia, major malaria vectors *An. funestus* s.s. and *An. gambiae* s.s. developed resistance to the dichloro-diphenyl-trichloroethane (DDT) and pyrethroids.^22,23^ The detection of cross-resistance to pyrethroids and organochlorines in 2010 prompted the Zambian National Malaria Control Programme to change its policy, transitioning from using long-lasting DDT to short-lived carbamates and organophosphates during the 2011 and 2012 IRS campaigns.^23^ Countrywide entomological monitoring conducted between 2012 and 2013 revealed high resistance to carbamates and pyrethroids in *An. funestus* s.s. and *An. gambiae* s.s. populations.^24,25^ As a result, in 2013, the National Malaria Control Programme stopped using carbamates and pyrethroids for IRS and adopted the organophosphate pirimiphos-methyl 300 CS as the Zambia’s IRS insecticide of choice, using it annually from 2014 through 2018 spray season.^23,26^

The Zambian MoH, in collaboration with its partners, has an ambitious goal of eliminating malaria nationally by 2021. To eliminate malaria in southern Zambia, mass distribution of LLINs and annual deployment of IRS are being scaled-up alongside expanded case management and mass drug administration (MDA) strategies. Mass drug administration with dihydroartemisinin–piperaquine (DHAp) is implemented at the community scale to clear *P. falciparum* infections and reduce human parasite reservoirs in Southern Province.^27^ To accelerate malaria elimination in low-transmission areas of Southern Province, MDA is effective when implemented in areas with good access to case management, strong surveillance, and universal coverage of vector control.^27^ *Anopheles funestus* is one of the major vectors of human malaria in much of Zambia, including parts of Southern Province.^28^ High parasite rates ranging from 6% to 20.0% have been reported in *An. funestus* from six provinces of Zambia.^25^ To reduce the vectorial capacity of *An. funestus* in the Southern Province of Zambia, the coverage of LLINs and IRS has increased at health facility catchment areas. With increased coverage of insecticide-based interventions, populations of *An. funestus* have developed resistance to pyrethroids, carbamates, and organochlorines in Zambia,^22,29–31^ as reported in almost every sub-Saharan African country.^16,30^ The resistance profiles observed in *An. funestus* from Zambia relate to the upregulation of cytochrome P450 oxidases.^22,25,29,31^ Presently, there is no case of kdr target-site mutations that has been detected in *An. funestus* populations in Zambia or elsewhere.^32^ However, data on the impact of universal coverage of vector control on insecticide resistance and its mechanisms in the malaria vectors along Lake Kariba of southern Zambia are limited. To ensure that insecticide choice is effective and sustainable for malaria vector control programs, the Global Plan for Insecticide Resistance Management encourages continuous monitoring of insecticide resistance and identification of resistance mechanisms in malaria major vector populations.^33^

In the context of locally aggressive malaria control and elimination, this study provides updated information on *An. funestus* susceptibility status to the four classes of insecticides, underlying metabolic resistance mechanisms, and *P. falciparum* sporozoite infection rates in Southern Province, Zambia. The results of this study also provide empirical evidence required to guide policy formulations and strategic implementation of sustainable insecticide resistance management strategies aimed at reducing malaria transmission in endemic areas of Southern Province.

## MATERIALS AND METHODS

### Study areas.

**Figure 1.**
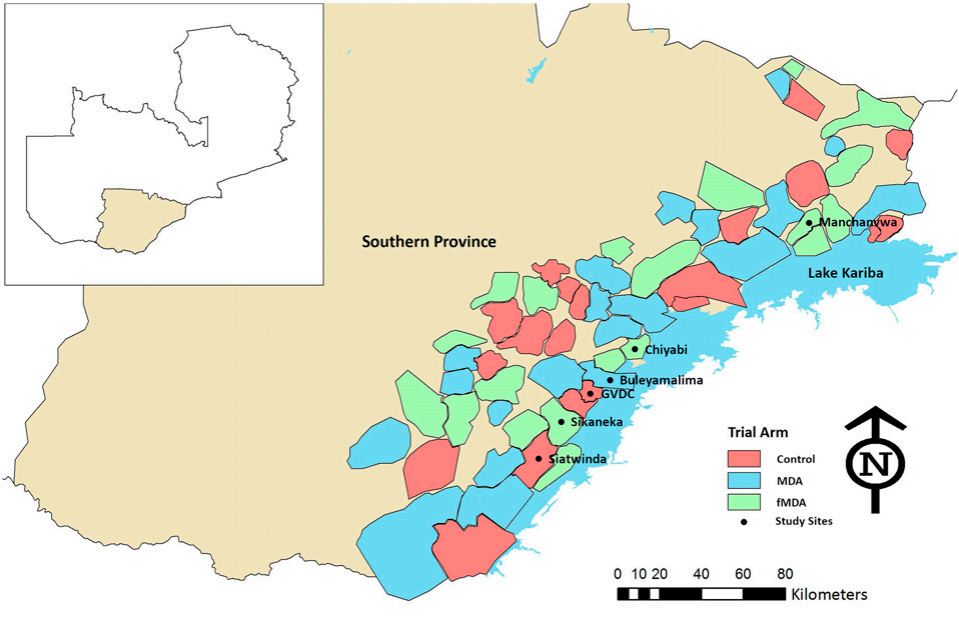
Map of Southern Province showing six study areas in Sinazongwe and Siavonga districts of Zambia, 2015.

The study was conducted in six study sites where an MDA trial was being implemented in Southern Province, Zambia (Figure 1). Details of the trial setup are given elsewhere.^27^ Entomological collections of mosquitoes in the study sites were performed from April 2015 to May 2015. The study sites were distributed along Lake Kariba in two districts, Siavonga and Sinazongwe. Mass distribution of pyrethroid-only LLINs, using PermaNet^®^ 2.0 (Vestergaard Frandsen, Saint Francois, Switzerland) and Olyset Net^®^ (Sumitomo Chemicals, Chuo, Japan), and IRS with the organophosphate pirimiphos-methyl (Actellic^®^ 300 SC, Syngenta, Basel, Switzerland) were the primary vector control tools used in the study areas. Scaled malaria case management by community health workers was supplemented with reactive case detection to areas surrounding index case households to identify additional infections.^34^ In addition, as part of the MDA trial, targeted MDA with DHAp was being conducted to clear malaria parasites from the local human populations in the study areas of Southern Province.^27^

The rainfall pattern in Southern Province is seasonal with the main rainy season starting in December and ending in April. Previous studies have shown that *Anopheles arabiensis* and *An. funestus* s.s. are the principal malaria vector species in Southern Province.^22,35,36^ The main economic activities practiced in the study areas of Southern Province are fishing and agriculture. To protect animals and crops from pests, farmers use different classes of insecticides in Southern Province as shown in Supplemental Table S1.

### Mosquito collections and rearing.

Indoor-resting blood-fed and gravid female *Anopheles* mosquitoes were collected in 20 randomly selected houses per site using a CDC Backpack Aspirator (John W. Hock Company) between 4:00 and 5:00 am. The collected adult mosquitoes were kept in BugDorm cages (Mega Science co. Ltd, Taichung, Taiwan) covered with moist cotton towels to support survival. The mosquitoes were later transported to the central laboratory at the National Malaria Control Centre in Lusaka for further processing. Gravid mosquitoes were induced to lay eggs in perforated Eppendorf tubes according to the protocol described by Morgan et al.^37^ After laying eggs, adult mosquitoes were removed and stored in well-labeled Eppendorf tubes. The eggs on the filter paper were immersed in paper cups filled with mineral water (Manzivalley^®^ Ltd, Natural Valley Limited, Chongwe, Zambia) for the development of aquatic mosquito stages. Rearing of mosquito larvae and pupae to F1s was conducted under climate-controlled standard laboratory conditions with a temperature range of 26 ± 2°C and 70–80% relative humidity.

### WHO susceptibility tests.

Bioassays were conducted using WHO tube kits to assess susceptibility or resistance of the F1 adult mosquitoes that emerged from those caught from the study sites. Four insecticide-impregnated papers were used: organochlorine (4% DDT), pyrethroid (0.05% deltamethrin), carbamate (0.1% bendiocarb), and organophosphate (0.25% pirimiphos-methyl), all procured from a WHO-collaborating center in Malaysia. Four batches of 20–25 unfed *An. funestus* females aged 2–5 days were exposed to each set of insecticide-treated papers for 60 minutes and maintained at 26 ± 2°C and 70–80% relative humidity in the insectary. The knockdown effects on *An. funestus* were monitored at 10-minute intervals over 60 minutes of exposure time. Final mortality of *An. funestus* was scored after a 24-hour post-exposure period, during which survivors were supplied with a 10% (w/v) sugar solution according to the WHO standard procedure.^38^ A negative control where wild-caught female *An*. *funestus* were exposed to untreated papers in the tube assays was used each time of testing. In the absence of a susceptible *An. funestus* strain for a positive control at the central laboratory, the susceptible colony of *An. gambiae* s.s. Kisumu strain was used to check the quality of insecticide-impregnated papers during bioassays.

### Piperonyl butoxide (PBO) synergist tests.

To understand the role of metabolic resistance in the *An. funestus* population, PBO, a synergist, was used in this study. Piperonyl butoxide inhibits the specific activity of P450 monooxygenases in insect populations.^39^ Subsamples of 20–25 unfed, 2- to 5-day-old F1 generation *An. funestus* were pre-exposed to 4% PBO-impregnated test papers for one hour. After pre-exposure to PBO, *An. funestus* populations were immediately exposed to 0.05% deltamethrin or 0.1% bendiocarb for an additional hour.^40^ One batch of 20–25 *An. funestus* were only exposed to 4% PBO without insecticide to serve as a control. After pre-exposure to PBO and either of the insecticides, *An. funestus* mosquitoes were transferred back to holding tubes and supplied with 10% sugar solution. Final mortality of both the controls and the *An. funestus* exposed to insecticides were scored after 24 hours.

### Parasite infection.

Sandwich ELISA was conducted on the head and thorax segments of dried random samples of wild-caught *An. funestus* that had laid eggs for resistance susceptibility bioassays. The detection of *P. falciparum* in populations of *An. funestus* was analyzed according to the protocol of Wirtz et al.^41^

### Species identification.

All field-collected mosquitoes were morphologically identified using the standard mosquito identification keys described by Gillies and De Meillon^42^ and Gillies and Coetzee,^43^and DNA was extracted from morphologically identified *An. funestus* mosquitoes from either the wings or legs of each mosquito sample.^44^ Sibling species of the *An. funestus* group were identified using a multiplex polymerase chain reaction (PCR) assay, and the PCR products were visualized using gel electrophoresis.^45^

### Data analysis.

Data were analyzed using Microsoft Excel^®^ (Microsoft Corporation, Redmond, WA) software. The prevalence of insecticide resistance in *An. funestus* was defined as per the standard WHO protocol by calculating mortality rate percentage 24 hours post-exposure. Effects of PBO on the mortality of *An. funestus* populations were determined by Student’s paired *t*-test. Binary logistic regression analysis using the generalized estimating equation (GEE) model accounting for clustering by using the replicates or batches of mosquitoes exposed in each WHO tube as the repeated measure was used to determine the difference in levels of susceptibility in *An. funestus* populations to insecticides between study sites. In this study, a *P*-value of ≤ 0.05 was considered to be significant.

### Ethical consideration.

Before mosquito collections, village meetings with community leaders and households were conducted to seek permission to trap mosquitoes in the study areas of Southern Province, Zambia.

## RESULTS

### Mosquito species composition.

A total of 2,516 indoor, blood-fed, and gravid mosquitoes were collected in 20 randomly selected houses in each study area using a CDC Backpack Aspirator, of which 76% (*n* = 1,912) were morphologically identified as *An. funestus* s.l. Giles, 16% (*n* = 402) as *An. gambiae* s.l. Giles, and the remaining 8% (*n* = 202) comprised other anophelines: *An. coustani* s.l. Laveran, *An. squamosus* Theobald, *An. rufipes* Gough, and *An. pretoriensis* Theobald*.*

### Species identification.

**Figure 2.**
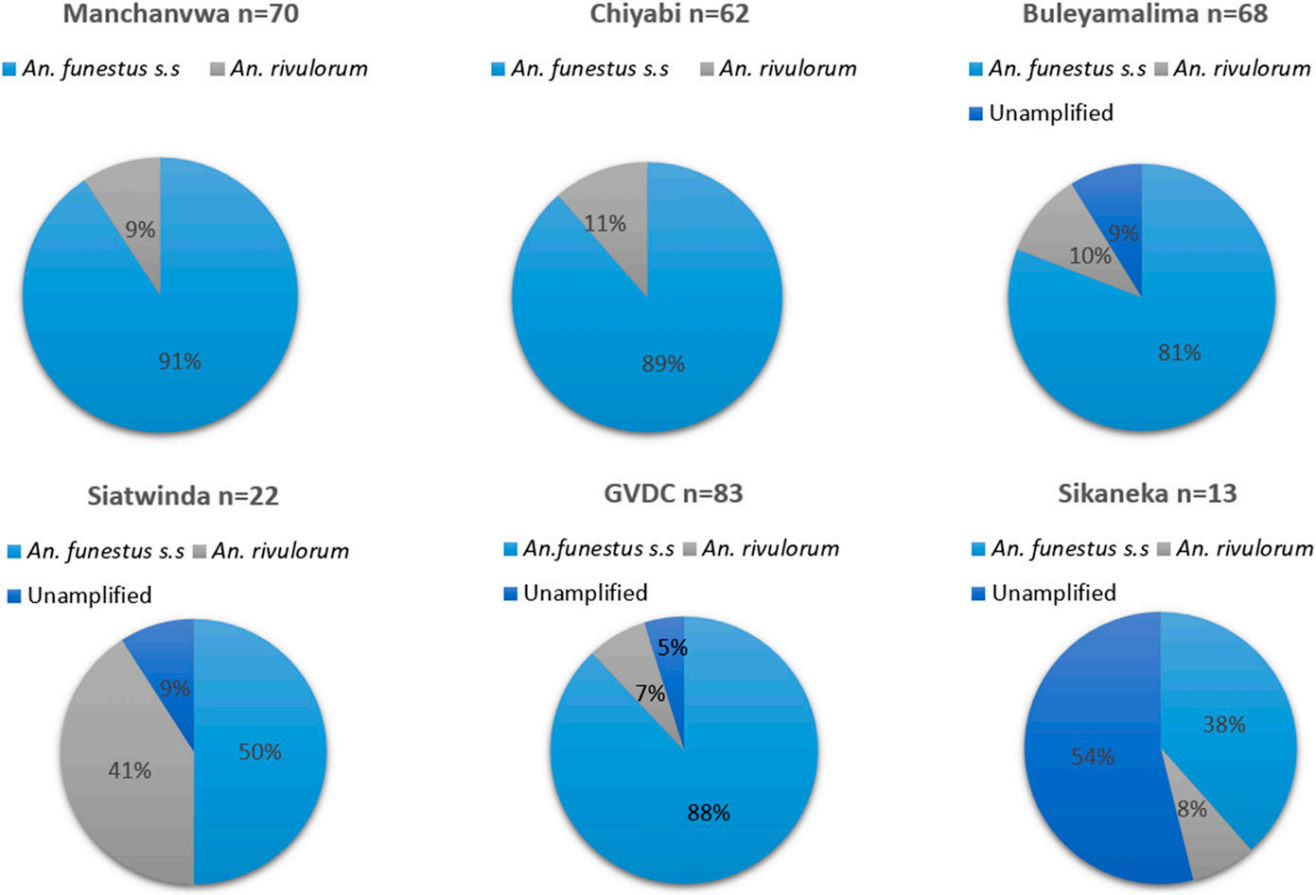
Vector species composition in six study areas of Southern Province, Zambia, 2015.

Polymerase chain reaction was performed on a random subsample (*n* = 310) of *An. funestus* that had laid eggs for insecticide resistance bioassays. Polymerase chain reaction results confirmed that 82.2% (*n* = 255) were *An*. *funestus* s.s., 11.6% (*n* = 36) *An. rivulorum*, and 19 samples failed to amplify as summarized in Figure 2.

### Insecticide susceptibility tests.

Results of WHO bioassays carried out on F1 generation *An. funestus* are summarized in Table 1. The mortality rate of *An*. *gambiae* s.s. Kisumu–susceptible laboratory-reared strain exposed to treated papers serving as a positive control was 100% (*n* = 200) for all tested insecticides. For the negative control, mortality rates of *An. funestus* were below 5% (Table 1). Based on the WHO criteria of 2013,^38^ mortality rates of *An. funestus* confirmed resistance to two insecticides, deltamethrin, and bendiocarb. Resistance to 0.05% deltamethrin (pyrethroid) was detected in *An. funestus* with mortality rates at 24 hours ranging from 13.8% (95% CI: 6.8–20.7) in the Gwembe Valley Development Centre (GVDC) to 52.0% (95% CI: 38.1–65.8) in Manchanvwa (Table 1). Exposure of *An. funestus* to 0.1% bendiocarb (carbamate) showed resistance with mortality rates at 24 hours ranging from 40.9% (95% CI: 30.9–50.8) in GVDC to 66.7% (95% CI: 55.3–78.0) in Manchanvwa (Table 1). Based on the estimates of the deltamethrin and bendiocarb models, there was a significant difference in the susceptibility of *An. funestus* to deltamethrin between study sites (GEE, *P* < 0.05), but no difference in study sites was observed with bendiocarb. By contrast, all populations of *An. funestus* were susceptible to 0.25% pirimiphos-methyl (organophosphate) and 4% DDT (organochlorine), with 100% mortality recorded at 24 hours post-exposure period across the study areas.

**Table 1:** WHO susceptibility bioassay test results on 2- to 5-day-old F1 *Anopheles funestus* s.l. in the Southern Province of Zambia, 2015

Study site	Deltamethrin (0.05%)	Bendiocarb (0.1%)	Pirimiphos-methyl (0.25%)	Dichloro-diphenyl-trichloroethane (4.0%)
	% Mortality (*n*)	% Mortality (*n*)	% Mortality (*n*)	% Mortality (*n*)
Buleyamalima	27.0 (100)	55.3 (94)	100 (44)	100 (54)
Gwembe Valley Development Centre	13.8 (94)	40.9 (93)	100 (100)	100 (99)
Chiyabi	28.0 (32)	47.0 (60)	100 (50)	100 (45)
Sikaneka	42.3 (26)	56.0 (50)	nd	100 (52)
Siatwinda	23.1 (52)	nd	100 (95)	100 (45)
Manchanvwa	52.0 (50)	66.7 (66)	100 (43)	100 (60)
Control*	2.5 (120)	3.8 (105)	4.9 (142)	4.5 (112)

*n* = number of *An. funestus* s.l. tested, % mortality: percentage mortality of *An. funestus* 24 hours post-exposure; nd = test not done.

* Control tests involved exposure of F1 *An. funestus* s.l. to untreated papers.

### Piperonyl butoxide bioassays.

Tables 2 and 3 show the role of metabolic resistance in populations of *An. funestus* using a synergist PBO. Pre-exposure of pyrethroid-resistant *An. funestus* to PBO increased the mortality rates to 100% against deltamethrin across the study sites (Table 2). The effects of PBO on pyrethroid-resistant *An. funestus* population were statistically significant (Table 2), excluding Sikaneka (paired sample Student’s *t*-test; *t* = 7.667, df = 1, and *P* = 0.0825). By contrast, PBO restored the full susceptibility status of *An. funestus* to bendiocarb in four study sites (Table 3) except in GVDC, where the mortality rates of resistant mosquitoes increased from 40.9% to 92.0% (paired sample Student’s *t*-test; *t* = 3.940, df = 4, and *P* = 0.029).

**Table 2:** Effects of piperonyl butoxide on the mortality of pyrethroid-resistant 2- to 5-day-old F1 *Anopheles funestus* s.l. mosquitoes in southern Zambia, 2015

Study site	Deltamethrin alone	PBO + Deltamethrin	Student’s *t*-test	
	% Mortality (*n*)	% Mortality (*n*)	*t*-value	*P*-value
Buleyamalima	27.0 (100)	100 (60)	24.33	0.001*
Gwembe Valley Development Centre	13.8 (100)	100 (50)	54.39	0.001*
Chiyabi	28.0 (32)	100 (40)	52.28	0.012*
Manchanvwa	52.0 (50)	100 (50)	12.00	0.053
Sikaneka	42.3 (26)	100 (50)	7.667	0.083
Siatwinda	23.1 (52)	100 (50)	19.98	0.032*

PBO = piperonyl butoxide.

* *P* < 0.05, paired Student’s *t*-test was used to compare *An. funestus* mortality with deltamethrin alone and when combined with PBO.

**Table 3:** Effects of piperonyl butoxide on mortality of carbamate-resistant 2- to 5-day-old F1 *Anopheles funestus* s.l. mosquitoes in southern Zambia, 2015

Study site	Bendiocarb alone	PBO + Bendiocarb	Student’s *t*-test	
	% Mortality (*n*)	% Mortality (*n*)	*t*-value	*P*-value
Buleyamalima	55.3 (94)	100 (40)	3.804	0.031*
Gwembe Valley Development Centre	40.9 (93)	92.0 (50)	3.940	0.029*
Chiyabi	47.0 (60)	100 (50)	7.341	0.018*
Manchanvwa	66.7 (66)	100 (44)	1.783	0.216
Sikaneka	56.0 (50)	100 (50)	2.750	0.222

PBO = piperonyl butoxide.

* P < 0.05, paired Student’s *t*-test was used to compare *An. funestus* mortality with bendiocarb alone and when combined with PBO.

### Parasite determination.

A total of 448 *An. funestus* mosquitoes were tested for the presence of the circumsporozoite protein of *P. falciparum* from their heads and thoraxes using ELISA. The parasite infection rates of *An. funestus* among the study areas ranged from 0% in Siatwinda (*n* = 48) and Sikaneka (*n* = 60) to 12.9% (95% CI: 5.0–20.7) in Chiyabi area (Table 4). The overall *P. falciparum* parasite prevalence in *An. funestus* populations was 5.8% (95% CI: 3.6–7.9) as summarized in Table 4.

**Table 4:** *Plasmodium falciparum* infection rates in *Anopheles funestus* s.l. in the Southern Province of Zambia, 2015

Study site	Number of mosquitoes tested	Number positive for *Plasmodium falciparum*	% Sporozoite rate
Buleyamalima	90	8	8.9
Gwembe Valley Development Centre	116	6	5.2
Chiyabi	70	9	12.9
Manchanvwa	64	3	4.7
Siatwinda	48	0	0.0
Sikaneka	60	0	0.0
Total	448	26	5.8

## DISCUSSION

The study demonstrated that *An. funestus* s.s. and *An. rivulorum* were the only members of the *An. funestus* group found in the six study areas of Southern Province after screening 310 samples with PCR. The subspecies of *An. funestus* group showed evidence of resistance to deltamethrin (a pyrethroid) and bendiocarb (a carbamate) but remained fully susceptible to DDT (an organochlorine) and pirimiphos-methyl (an organophosphate) in Southern Province. Pre-exposure of *An. funestus* populations to PBO restored the susceptibility of pyrethroid and carbamate resistance observed in five study areas. Nonetheless, carbamate resistance was partially restored in populations of *An. funestus* from GVDC with 92% mortality 24 hours post-exposure. The results of the study revealed high variation in the *P. falciparum* infection rates in *An. funestus* across the six study areas.

Pyrethroid and carbamate cross-resistance reported in southern Zambia mirrors the experience in neighboring Mozambique,^46,47^ Malawi,^48,49^ and Zimbabwe^29,50^ and suggests a regional vector control challenge. Detection of cross-resistance in the malaria vector *An. funestus* is of major concern and limits insecticide selection choice for resistance management in southern Zambia. The absence of cross-resistance between pyrethroid and DDT in *An. funestus* across the study areas might suggest that a kdr-type of target-site resistance mechanism has not been selected for by the two insecticides at the time of the study. The complete susceptibility of *An. funestus* to pirimiphos-methyl and DDT observed in the study areas suggest that the two insecticides are still effective against these species and could be used as alternatives to pyrethroids and carbamates for the effective scale-up of IRS operations in southern Zambia. Nonetheless, DDT cannot be used for IRS operations in areas where *An. gambiae* s.s. dominate largely because of high levels of resistance documented within this species.^24^ To safeguard the limited number of insecticide classes available for vector control, the WHO Global Plan for Insecticide Resistance Management in Malaria Vectors encourages malaria-endemic countries to develop insecticide resistance management plans based on the local evidence.^33,51^ Adoption of effective insecticide resistance management strategies depends on assessing susceptibility of malaria vector populations to classes of insecticides used for public health. Previous studies conducted in Eastern Province, Zambia, have shown that rotations of non-pyrethroid insecticide classes (such as organophosphate: pirimiphos-methyl) in IRS areas reduced the intensity of resistance of mosquito populations to pyrethroid molecules.^23^

Detection of pyrethroid and carbamate cross-resistance in *An. funestus* populations in the study areas of Southern Province could be attributed to a number of factors ranging from rapid scale-up of insecticide treated nets (ITNs)/LLINs and IRS, and gene flow to use of pesticide in agriculture.^48,52,53^ Indeed, coverage of pyrethroid-impregnated ITNs increased from 46.9% in 2006 to 77.8% in 2015 across the 13 districts of Southern Province and might contribute to the pyrethroid resistance profiles being reported in the study areas. Furthermore, review of insecticides used for agriculture (Supplement Table S1) shows that a number of carbamates are used in the study areas for agriculture^54,55^ and could be responsible for selecting carbamate resistance in *An. funestus* populations.^52^ In addition, the observed carbamate resistance in *An. funestus* within the study areas could be related to pyrethroid cross-resistance mediated by metabolic mechanisms as previously reported from Zimbabwe, Malawi, and South Africa.^50,56,57^

Understanding the role of the metabolic resistance mechanism in malaria vectors is critical for the effective implementation of insecticide-based vector interventions. In this study, pre-exposure of *An. funestus* populations to PBO restored susceptibility of pyrethroid and carbamate in the study areas, suggesting that P450 monooxygenases play a role in the resistance phenotype. By contrast, the partial recovery of *An. funestus* susceptibility after PBO pre-exposure to carbamates in a highly mechanized agricultural area of GVDC highlights the possible presence of other metabolic enzyme groups in the study areas besides P450 monooxygenases. The study findings further suggest that PBO-impregnated bednets (PermaNet^®^ 3.0 [Vestergaard Frandsen, Saint Francois, Switzerland] and Olyset Net Plus^®^ [Sumitomo Chemicals, Chuo, Japan]) could counter such resistance mechanisms and aid malaria reduction in Southern Province, Zambia. The role of P450 monooxygenases in causing high pyrethroid resistance in *An. funestus* and *An. gambiae* s.l. populations has been previously reported in Zambia,^22,25,30,31^ and the information guided the National Malaria Control Programme to select effective resistance management strategies for malaria control and elimination.

Assessing vector competence is usually achieved through finding an infectious stage of the malaria parasite (the sporozoite) in the salivary glands of a mosquito. High *P. falciparum* sporozoite infection rates in *An. funestus* were recorded in four study areas: Manchanvwa, GVDC, Buleyamalima, and Chiyabi, whereas no sporozoites were found in mosquitoes from Sikaneka and Siatwinda. The detection of parasite infection rates of 5.8% in populations of *An. funestus* from these four study areas of southern Zambia highlight its potential role in malaria transmission and requires an effective integrated vector control program. These study findings agree with previous studies that documented a parasite rate of 10% in 2013 in *An. funestus* in Southern Province.^25^ By contrast, the absence of malaria parasites in *An. funestus* from Sikaneka and Siatwinda could be associated with high spray coverages exceeding 85% during the 2014 IRS campaigns compared to other study areas. The findings are consistent with previous studies which demonstrated the impact of IRS on reducing sporozoite infection rates of *An. funestus* in the presence of pyrethroid resistance in Bioko Island^58^ and Mozambique.^59^ However, because of the fact that only 448 samples were analyzed, more studies are required to better assess infectivity rates across the sites. Despite detecting *An. rivulorum* in the study areas, its epidemiological role in malaria transmission remains unknown in southern Zambia. Nonetheless, previous studies conducted in Tanzania^60^ and Kenya have detected *P. falciparum* in *An. rivulorum*.^61^

Our study had some limitations. The small sample size of *An. funestus* mosquitoes used for the determination of WHO susceptibility and resistance bioassays provided information on a limited scale to infer the presence but not the level of resistance in the study areas. Wider scale studies with adequate sample size from not only Southern Province but also from other sites across the country are needed to better inform the insecticide resistance profile of vectors in Zambia. We also had insufficient resources (including a lack of PCR primers) to analyze all specimens to confirm species identities, and so not all samples that were exposed to insecticides underwent PCR to determine the sibling species of the *funestus* group. Although most appeared to be *An. funestus* s.s., we cannot confidently associate the resistant phenotypes seen with that species alone. Furthermore, resources were limited to perform molecular genotyping to characterize resistance mechanisms in the populations of *An. funestus* in the study areas. There is a need for further studies to determine more comprehensively the insecticide resistance mechanisms present in the study areas to guide the technical advisory committee of insecticide resistance management at the national scale.

Detection of pyrethroid and carbamate resistance in *An. funestus* populations provides a platform for increased insecticide resistance monitoring along Lake Kariba. To prevent the development of metabolic resistant mosquitoes and adequately reduce the underlying vectorial capacity in areas targeted for malaria elimination in southern Zambia, adoption of an effective integrated vector management strategy based on local empirical evidence will be needed. An integrated vector management strategy focused on strengthening collaboration ties between its public health and agricultural sectors, encouraging rotational use of IRS insecticides, integrating PBO LLINs and community-based larviciding, house screening, and enhancing insecticide resistance monitoring should be considered. New insecticides for both IRS (clothianidin, chlorfenapyr, and Fludora Fusion) and LLINs (Interceptor G2 and Royal Guard) have been developed in recent years, and they may be valuable additions in extending Zambia’s vector control arsenal as the country seeks malaria elimination by 2021.

## Supplemental table

Supplemental materials
