# Supplementary material for: Pyrethroid and Carbamate Resistance in Anopheles funestus Giles along Lake Kariba in Southern Zambia
Source: Am J Trop Med Hyg. 2020 Jul 2;103(2 Suppl):90–7. doi: 10.4269/ajtmh.19-0664 (PMC7416976; doi:10.4269/ajtmh.19-0664)
Supplement: Supplementary file 1 [file tpmd190664.SD1.docx]

**Supplemental Table S1:** Common insecticides used for agriculture sector in Southern Province of Zambia (sources: Ministry of Agriculture and Livestock, 2011; Ministry of Finance, 2013).

| Class of Insecticide | Trade Name | Insecticide Name | Use of insecticides |
| --- | --- | --- | --- |
| Carbamates | Butacarly | Butacarb | Sheep dip, control of ticks and mites |
|  | Carbaryl, Sevin, Carbax | Carbaryl | Control of poultry mites |
|  | Baygon, Bayopet powder | Propoxur | Control ectoparasites, flies and cockroaches |
|  | Ficam | Bendiocarb | Control poultry for mites and flies |
| Pyrethrum  and  pyrethroids | Pyrethrum | Pyrethrins | Control of flies, ectoparasites on cattle and small ruminants |
|  | Paracide | Alphamethrin | Control of ticks and flies on cattle and small ruminants, cotton, bollworms. |
|  | Cyrux, Cyperdip, Cypernel, Sectar | Cypermethrin | Control of ticks and flies on cattle and small ruminants, tomato moth, fruit fly, white fly, spotted stem borer, brown leaf beetle. |
|  | ICON, Tata Reeva, | Lambdacyhalothrin | Control of housefly, cockroaches, ball worms, army worms, stoke borers, house fly, sucking insects, |
|  | K-Orthrine, Deltarex, Decatix, Butox, Decis. | Deltamethrin | Control of ticks and tsetse fly on cattle, bollworms, white fly, spotted stem borer and phorid fly, cockroaches, red flour beetle, rice moth. |
|  | Deadline | Flumethrin | Control of ticks and tsetse fly on cattle |
|  | Ambush, Coopex | Permethrin, | Control of flies, sweet potato weevils, termites, black beetle, aphids and diamond back moth. |
| Organophosphates | Nexagon, Nexagran | Bromophos –ethyl | Control of ticks on cattle and small ruminants |
|  | Supona, Supona super, Supona aerosol. | Chlorfernvinphos | Control of ticks, mites and flies on Cattle and sheep |
|  | Asunto, Baymix | Chlorpyrifos | Control of ticks, mites, fleas on cattle, sheep, poultry, white fly, black beetle, cut worms and termites. |
|  | Doom, Vapona, Dedevap, Nuvan | Dichlorvos | Control of Flies, mites and lice in animal houses, caterpillars, red pumpkin beetle, brown plant hopper. |
|  | Ronnel, Lanokil | Fenchlorphos | Control of ectoparasites on cattle, sheep, goats |
|  | Sumithion, Decofen | Fenitrothion | Control of flies, fleas, lice and coreid bugs. |
|  | Bacdip, Quintiophos | Oxinthiophos | Cattle dip for resistant ticks on cattle |
|  | Benzphos, Ambacide | Phosalone | Tick and fly control on cattle and small ruminants |
|  | Actellic, Blex | Pirimiphos methyl | Fly control on small ruminants and grain weevils. |
|  | Malathion | Malathion | Control of tobacco caterpillar, white fly, beetles, mites, stem borer. |
